# Heparin antagonizes cisplatin resistance of A2780 ovarian cancer cells by affecting the Wnt signaling pathway

**DOI:** 10.18632/oncotarget.18738

**Published:** 2017-06-28

**Authors:** Daniel Bastian Pfankuchen, Fabian Baltes, Tahira Batool, Jin-Ping Li, Martin Schlesinger, Gerd Bendas

**Affiliations:** ^1^ Pharmaceutical Institute, Rheinische Friedrich-Wilhelms-University Bonn, Bonn, Germany; ^2^ Department of Medical Biochemistry and Microbiology, SciLifeLab, University of Uppsala, Uppsala, Sweden

**Keywords:** tinzaparin, cancer, chemoresistance, Wnt, cisplatin

## Abstract

Low molecular weight heparin (LMWH), the guideline based drug for prophylaxis and treatment of cancer-associated thrombosis, was recently shown to sensitize cisplatin resistant A2780cis human ovarian cancer cells for cisplatin cytotoxicity upon 24 h pretreatment with 50 μg × mL^−1^ of the LMWH tinzaparin *in vitro*, equivalent to a therapeutic dosage. Thereby, LMWH induced sensitization by transcriptional reprogramming of A2780cis cells via not yet elucidated mechanisms that depend on cellular proteoglycans. Here we aim to illuminate the underlying molecular mechanisms of LMWH in sensitizing A2780cis cells for cisplatin. Using TCF/LEF luciferase promotor assay (Top/Flash) we show that resistant A2780cis cells possess a threefold higher Wnt signaling activity compared to A2780 cells. Furthermore, Wnt pathway blockade by FH535 leads to higher cisplatin sensitivity of A2780cis cells. Glypican-3 (GPC3) is upregulated in A2780cis cells in response to LMWH treatment, probably as counter-regulation to sustain the high Wnt activity against LMWH. Hence, LMWH reduces the cisplatin-induced rise in Wnt activity and TCF-4 expression in A2780cis cells, but keeps sensitive A2780 cells unaffected. Consequently, Wnt signaling pathway appears as primary target of LMWH in sensitizing A2780cis cells for cisplatin toxicity. Considering the outstanding role of LMWH in clinical oncology, this finding appears as promising therapeutic option to hamper chemoresistance.

## INTRODUCTION

Malignant tumor diseases induce an upregulation of blood coagulation by a functional interlinkage that has first been described more than 150 years ago, referred to as Trousseau syndrome [1]. Tumor patients display a 4- to 6-fold higher incidence to suffer from venous thrombotic events, and thus thrombosis turns out to be the second most common cause of death in oncology. Consequently, antithrombotic prophylaxis is an important component in the therapeutic regimens of cancer patients. According to clinical guidelines for antithrombotic prophylaxis or treatment of patients in oncology, low molecular weight heparin (LMWH) is the drug of choice [2].

There is an ongoing and still controversial discussion whether LMWH can induce more than circumvention of thrombosis in cancer diseases. A retrospective evaluation of clinical data referred to a survival benefit of LMWH treated cancer patients [3], which was confirmed for patient subgroups in a number of prospective clinical trials [4, 5]. Nevertheless, other clinical trials failed to confirm statistically an impact of heparin on survival rates [6, 7]. However, there is accumulating evidence from preclinical studies that heparin has a capability to inhibit the process of metastatic tumor spread [8]. Obviously, triggered by the glycosaminoglycan (GAG) structure of heparin, LMWHs can interfere at various stages of the metastatic cascade and thus attenuates tumor cell adhesion, growth factor activity, angiogenesis, enzymatic heparanase activity and thrombin-induced prometastatic signaling [9, 10].

However, little is known whether LMWH affects the efficiency of cytostatic treatment of solid tumors, which is explicitly addressed in only few studies. Patients with pancreatic cancer [11] or small cell lung cancer were shown to benefit from a combination of the cytostatics with LMWH [12–14]. A reduced thrombosis tendency of tumors by heparin treatment was supposed to enhance the access of drugs to the tumor tissue. Though, the major obstacle in clinical cancer therapy remains the rapid development of tumor cell resistance against cytostatic toxicity, referred as chemoresistance [15]. Cisplatin, the standard drug for treatment of e.g. ovarian malignancies is frequently affected by multiple mechanisms of chemoresistance [16]. However, pharmacological approaches to target chemoresistance are rare.

We recently reported the surprising finding that a therapeutic dose of the LMWH tinzaparin reversed the cisplatin resistance of A2780cis human ovarian cancer cells *in vitro* to the level of A2780 cells [17]. Although the molecular mechanisms of this activity remain unknown, heparitinase susceptibility of cellular resistance and LMWH efficiency referred to a heparan sulfate proteoglycan (HSPG)-triggered resistance mechanism as target for LMWH. Here, we elucidate the underlying mechanisms of A2780cis cisplatin resistance to understand the sensitizing LMWH activity.

Referring to HSPG-dependency that probably triggers tumor cell resistance, we proposed three potential pathways: i) Heparanase, the sole endoglycosidase in mammals modulates HSPGs and is considered as a bad prognostic marker in multiple malignant diseases [18], which also triggers resistance [19]. This enzyme is known to be a target for LMWH in clinical trials [20]. ii) Syndecans are HSPGs that act as co-receptors for integrins in binding extracellular matrix (ECM) substrates and thus promote tumor resistance. Cell adhesion mediated drug resistance (CAM-DR) is clinically evident by a relapse after initial remission, known as “minimal residual disease” [21]. iii) Finally, canonical Wnt signaling pathway appears as attractive target which is known to be responsible for e.g. tumor cell resistance [22, 23] and is controlled in its activity by various HSPGs, most likely the family of glypicans [24]. Among several other pathways triggering malignancy, which were also dependent on HSPGs, such as FGF-signaling pathway, we explicitly focused our activities on the Wnt signaling pathway, since our gene array data revealed that a tinzaparin treatment of A2780cis cells induces a massive change in signaling activity, and Wnt signaling appears as the most deregulated one.

Considering the canonical Wnt signaling pathway, in the deactivated state cytoplasmatic localized β-catenin is phosphorylated by a destruction complex, which consists of APC (adenomatous polyposis coli), Axin, CK1α (casein kinase 1 alpha) and GSK3-β (glycogen synthase kinase 3 beta). After phosphorylation, β-catenin is marked for proteasomal degradation mediated through β-TrCP (beta-transducin repeat containing E3 ubiquitin protein ligase). Wnt signaling is activated through Wnt proteins, which bind to a transmembrane receptor complex, which consists of Fzd (Frizzled) and the co-receptor LRP-5/6. This leads to a recruitment of Axin and Dvl (Dishevelled 1) to the membrane and therefore inactivating the β-catenin destruction complex. As a consequence, β-catenin accumulates in cytoplasma and enters the nucleus, leading to an association with TCFs and therefore activation of transcription of Wnt signaling target genes [25–27]. One of the transcription factors is TCF-4 (TCF7L2), which represents a key protein in Wnt signaling. Strong evidence exists that TCF-4 driven Wnt signaling activity leads to initiation and progression of human carcinogenesis, mediated through overexpression of oncogenes like c-MYC and Cyclin D1 [28–33].

Here we can show that the Wnt signaling pathway is one of the dominant processes to induce cisplatin resistance in A2780cis cells, which appears as the primary target for tinzaparin in reversing the resistance. The elucidation of a functional axis of LMWH and Wnt pathway for overcoming chemoresistance sheds a new light on LMWH application in oncology.

## RESULTS

### HSPG expression pattern of A2780 and A2780cis cells

**Figure 1:**
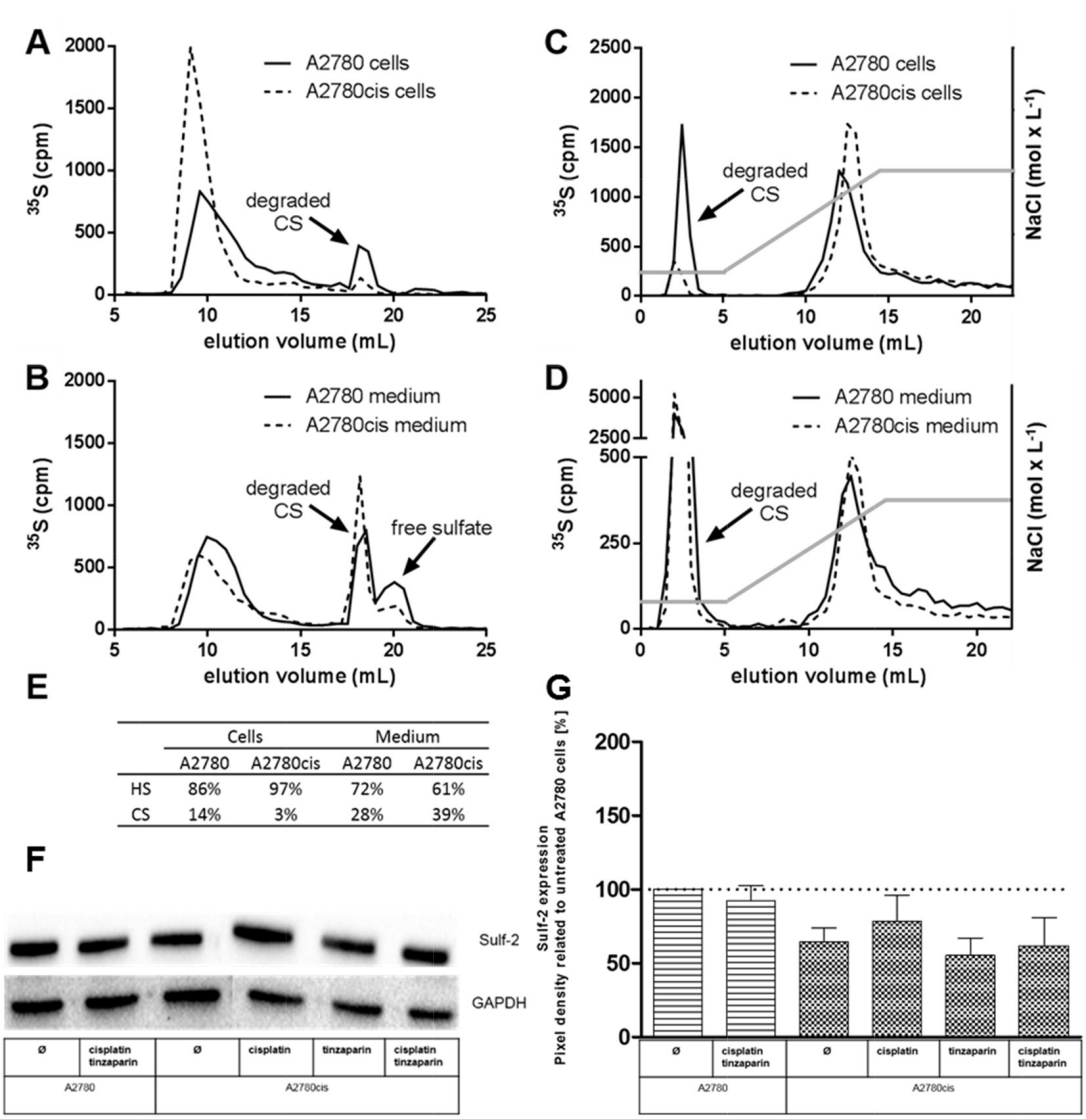
Characterization of GAG molecular structure of A2780 and A2780cis cells Metabolically labeled GAG isolated from A2780 and A2780cis cells **(A, C)** and their cultivation medium **(B, D)** were analyzed. **(A, B)** Size exclusion chromotography analysis of the ^35^S-labeled samples indicates higher HS fractions in A2780cis cells. **(C, D)** Ion exchange chromatography on analysis of the ^35^S-labeled samples eluted by a linear NaCl gradient of 0.25 - 2 mol × L^−1^ (indicated by the grey line) display a higher charge density of the A2780cis HS. The degraded CS saccharides are indicated. The table in **(E)** shows the proportion of HS and CS in cell and medium fractions. Data are average of two independent experiments. **(F)** Expression of Sulf-2 in A2780 and A2780cis cells without pretreatment, or after a 24 h tinzaparin preincubation before cisplatin addition. Expression was analyzed 72 h after cisplatin treatment by immunoblotting. Data are shown as blot for one representative experiment **(F)** and as relative expression by pixel density measurements related to untreated A2780 cells **(G)**. Data from three independent experiments.

Sensitization of A2780cis cells for cisplatin cytotoxicity by the LMWH tinzaparin was shown to be dependent on an intact HSPG interactome at the cell surface, illustrated by heparitinase susceptibility of resistance and sensitization [17]. To obtain an insight into the mode of action of LMWH, we initially analyzed the expression pattern of HSPG with respect to differences in heparan sulfate (HS) chain length and degree of sulfation between A2780 and A2780cis cells. Examination of metabolically ^35^S-labeled glycosaminoglycans (GAG) revealed that the HS expressed in A2780cis cells are longer in chain length (Figure 1A) and display a higher degree of sulfation (Figure 1C) than that in A2780 cells, which is in lower extent also visible in the medium fraction (Figure 1B and 1D). Interestingly, the cell medium contains higher amounts of chondroitin sulfate (Figure 1E) than represented by the cell bound fraction. However, HS comprised the dominant fraction of GAG compared to chondroitin sulfate (CS) in both cell lines, this ratio is even more increased in the A2780cis cells (Figure 1E). Furthermore, A2780cis cells express slightly lower levels of the sulfatase Sulf-2 than A2780 cells, which were also not significantly affected by tinzaparin or cisplatin treatment (Figure 1F, 1G).

### Cell adhesion mediated resistance appears not relevant in A2780cis cells

**Figure 2:**
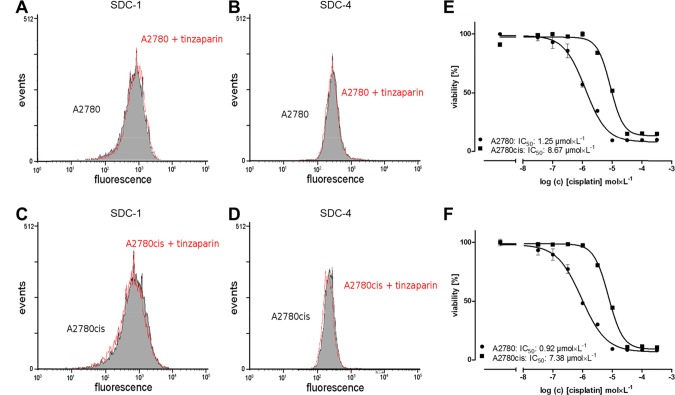
Analysis of cellular syndecan expression pattern and the impact of ECM on resistance Syndecan-1 (left panel) and -4 (right panel) in tinzaparin treated A2780 **(A, B)** and A2780cis **(C, D)** cells. The expression was analyzed by flow cytometry using specific antibodies indicating no impact of tinzaparin on syndecans. Experiments were performed in triplicates. Determination of cytotoxicity 72 h after addition of cisplatin displayed as IC_50_ in A2780 (circles) and A2780cis (squares) cells by MTT assays. The IC_50_ for cisplatin **(E)** and in combination with collagen coated wells **(F)** are indicated for a representative experiment. MTT assays were performed in triplicates.

Syndecan-1 (SDC-1) and syndecan-4 (SDC-4), both alone or as co-receptors for integrins are crucially involved in the phenomenon of environmental-mediated drug resistance of tumors [34], which appears as probable target for LMWH in light of heparin integrin binding or competition with syndecan GAG moieties. To investigate the potential role of CAM-DR in A2780cis cells as target for LMWH we analyzed SDC-1 and SDC-4 expression in response to a “sensitizing” 24 h preincubation with 50 μg × mL^−1^ tinzaparin. Neither A2780 cells (Figure 2A and 2B) nor A2780cis cells (Figure 2C and 2D) show a remarkable change in expression of SDC-1 or SDC-4 related to tinzaparin preincubation. Furthermore, cisplatin cytotoxicity measurements of both cell lines with an incubation on collagen as an ECM simulation for integrin binding has no impact on the sensitivity to cisplatin, when compared to plain surface cultivation (Figure 2E, 2F). Consequently, CAM-DR can be excluded as a potential mechanism of chemoresistance

### Impact of heparanase on A2780cis resistance

**Figure 3:**
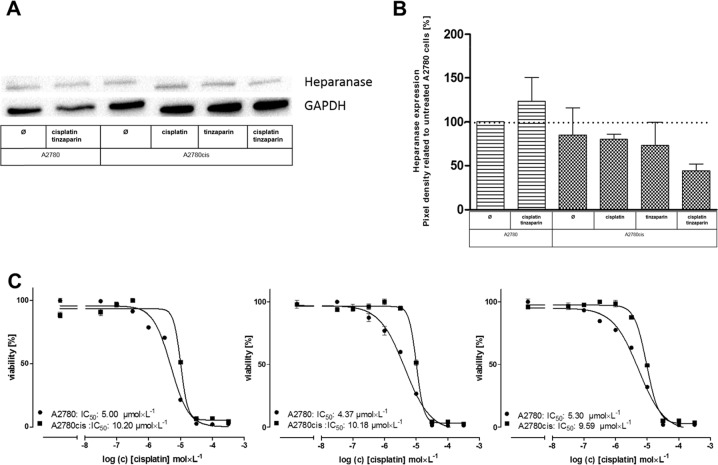
**(A)** Expression of heparanase in A2780 and A2780cis cells preincubated 24 h with tinzaparin before cisplatin addition, with cisplatin or both. Expression of heparanase was analyzed 72 h after cisplatin treatment by immunoblotting using a specific antibody and shown as blot for one representative experiment and as relative expression by pixel density measurements related to untreated A2780 cells, summarized in **(B)** from three independent experiments. **(C)** Determination of cytotoxicity 72 h after addition of cisplatin displayed as IC_50_ in A2780 (circles) and A2780cis (squares) cells by MTT assays. The IC_50_ for cisplatin (left) and in combination with a preincubation of 5 (middle) and 10 μmol × L^−1^ (right) roneparstat 5 h before addition of cisplatin are indicated in the figure for a representative experiment. MTT assays were performed in triplicates.

Heparanase, the sole endoglycosidase in mammals is considered as a bad prognostic marker in different malignancies, based on its capacity to cleave HSPGs, to remodel ECM and to turn tumor cells into chemoresistance [19]. To elucidate whether the known interference of LMWH with heparanase activity is a valid target for A2780cis resistance, we compared heparanase expression in A2780 and A2780cis cells related to cisplatin or combined cisplatin and tinzaparin treatment. It becomes evident (Figure 3A) that heparanase seems to be slightly lower expressed in A2780cis than in A2780 cells, which could explain the somewhat higher size of the HS chain length in A2780cis cells (Figure 1A). Tinzaparin in combination with cisplatin induces a slight upregulation in A2780 cells, while tinzaparin and cisplatin as single treatments hardly affect the heparanase expression in A2780cis cells. However, they induced a downregulation, when combined in A2780cis cells. This behavior makes a supposed role of heparanase in A2780cis resistance unlikely. As a functional confirmation, we applied the established heparanase inhibitor roneparstat which has no effect on the sensitivity of the cells to cisplatin (Figure 3B).

### Gene expression data refer to Wnt signal deregulation

**Figure 4:**
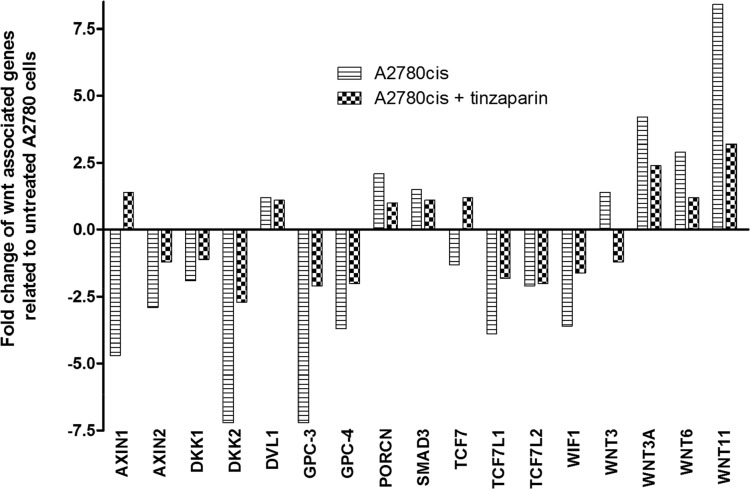
Analysis of Wnt-associated genes in untreated (stripes) and 24 h tinzaparin treated (squares) A2780cis cells Fold change of transcription was normalized to untreated A2780 cells.

The whole genome array data of A2780cis cells after 24 h LMWH pretreatment in our recent study [17] referred to the Wnt pathway as one of the most probably deregulated signaling pathways in the GO term analysis. Following this indication we started a detailed view on the mRNA levels of Wnt pathway associated components in A2780cis cells and their regulation by LMWH, compared to A2780 cells as baseline level (Figure 4). Interestingly, potential Wnt pathway inhibitors, such as Axin1 and 2 or members of the dickkopf family DKK1 and DKK2 display a strong downregulation in A2780cis cells, but these attenuated mRNA levels were obviously reversed by cellular tinzaparin pretreatment. In line with this, even the upregulated mRNA of Wnt ligands (Wnt 3, 3A, 6 and 11) is attenuated by tinzaparin. In contrast to these findings, which indicate an intensified Wnt activity in A2780cis cells compared to A2780, the mRNA data of the transcription factors TCF-3 and 4 (TCF7L1 and 2) are reduced which provokes a further attention to check that at protein level. Interestingly, the proteoglycan glypican-3 (GPC3) which has been shown to be directly involved in Wnt signaling [35], shows remarkable downregulation at the mRNA level, which appears contradicting with Wnt activity and necessitates further investigation.

### Glypican-3 and 4 expression and deregulation by LMWH

**Figure 5:**
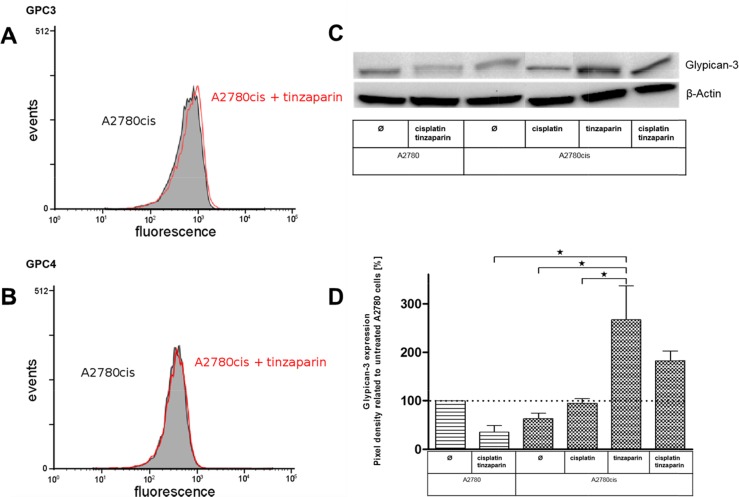
Analysis of the HSPGs glypican-3 **(A)** and glypican-4 **(B)** in A2780cis cells with a 24 h tinzaparin preincubation (50 μg × mL^−1^). The expression was analyzed by flow cytometry using specific antibodies. Experiments were performed in triplicates. **(C)** Expression of glypican-3 in A2780 and A2780cis cells with a 24 h tinzaparin preincubation before cisplatin addition. Expression was analyzed 72 h after cisplatin treatment by immunoblotting using a specific antibody and shown as blot for one representative experiment and as relative expression by pixel density measurements related to untreated A2780 cells **(D)**. Data from two independent experiments.

To further address the role of GPC3 and GPC4 in A2780cis cells and their potential role for resistance formation via Wnt signaling we checked their expression levels and their deregulation by a 24 h preincubation with 50 μg × mL^−1^ tinzaparin by flow cytometry. It becomes evident that GPC3 in A2780cis cells, compared to A2780 cells (data not shown) display a lower expression, which is not remarkably deregulated by tinzaparin (Figure 5A). GPC4 is also not affected by tinzaparin (Figure 5B).

However, overcoming of cisplatin resistance by tinzaparin was detected after 72 h based on the MTT cytotoxicity protocol. Therefore we investigated by western blot how GPC3 is affected by cisplatin and / or tinzaparin after this time frame. In agreement with the mRNA data and flow cytometry findings, A2780cis cells display a lower expression of GPC3 than A2780cells, when both cell lines are untreated, but cisplatin seems to provoke GPC3 in A2780cis cells. Even more interesting, tinzaparin causes a massive upregulation of GPC3 in A2780cis cells (Figure 5C).

### Cisplatin resistance in A2780cis cells is susceptible for Wnt pathway inhibition

**Figure 6:**
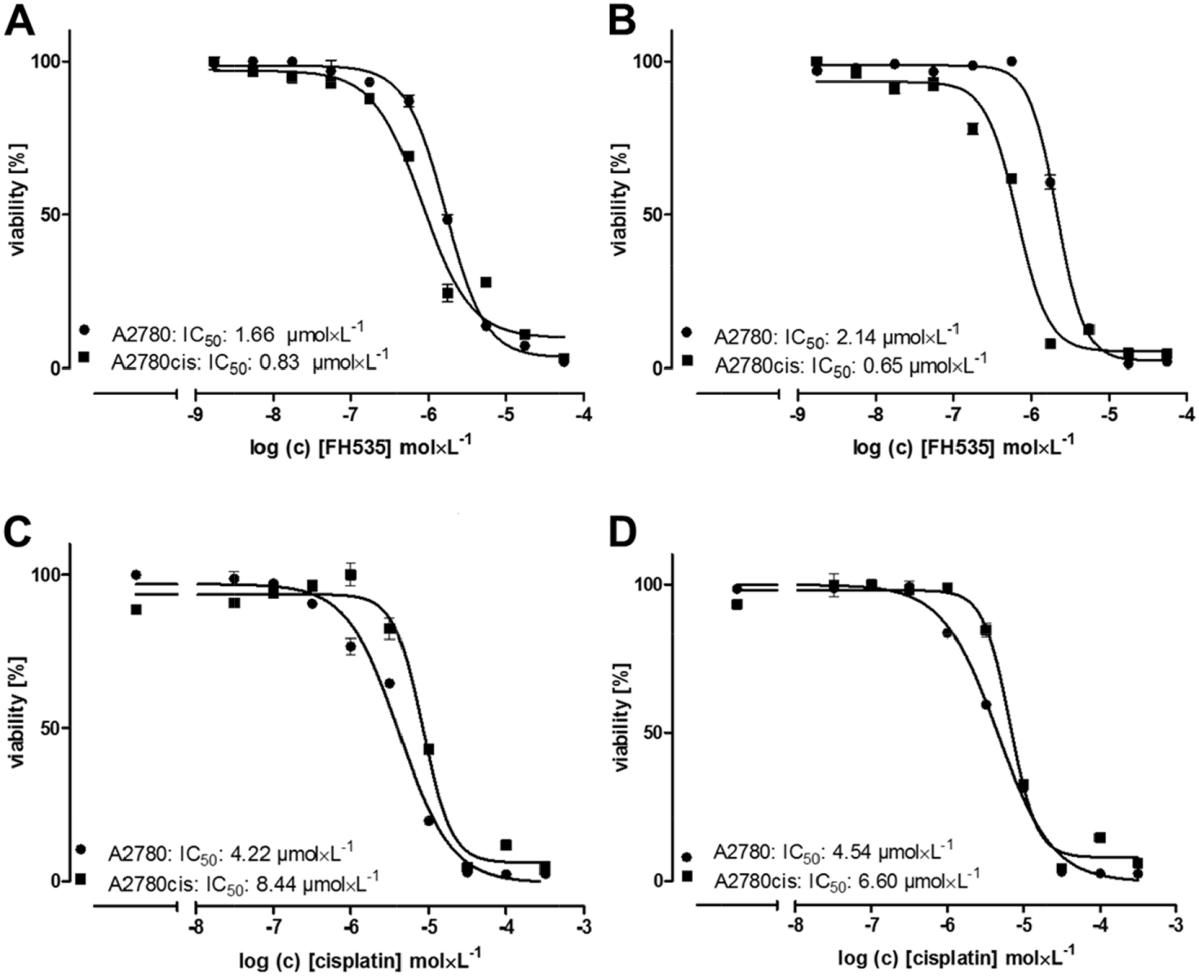
Cytotoxicity of the Wnt pathway inhibitor FH535 alone and in combination with cisplatin Data are displayed as IC_50_ in A2780 (circles) and A2780cis (squares) cells by MTT assays. The IC_50_ for FH535 **(A, B)**, cisplatin **(C)** and cisplatin in combination with a preincubation with 0.5 μmol × L^−1^ FH535 1.5 h before addition of cisplatin **(D)** are indicated in the figure for a representative experiment. Cells were seeded in a count of 20,000 **(A)** and 40,000 **(B, C, D)** 24 h before treatment with FH535 and cisplatin. MTT assays were performed in triplicates.

Next we aimed to investigate whether a potentially higher Wnt activity can functionally be reflected in A2780cis cells referring to cisplatin cytotoxicity. In doing so, we applied the Wnt pathway inhibitor FH535, which is a small molecule inhibitor for the Wnt β-catenin pathway [36]. First we checked the sensitivity of A2780 and A2780cis cells to increasing FH535 concentrations (Figure 6A and 6B). It is noteworthy to point out that A2780cis cells, at two different cell densities show a higher susceptibility towards FH535 cytotoxicity than A2780 cells. To further investigate how Wnt inhibition affects cisplatin cytotoxicity, we applied FH535 at a sub-toxic dose of 0.5 μmol × L^−1^ and performed a cisplatin cytotoxicity (MTT) assay in A2780 and A2780cis cells. While FH535 has no impact on the cisplatin efficiency in A2780 cells showing nearly identical IC_50_ values (Figure 6C and 6D), the cisplatin resistance in A2780cis cells is remarkably reduced in presence of FH535.

### Wnt activity in A2780cis and A2780 cells and effects of tinzaparin

**Figure 7:**
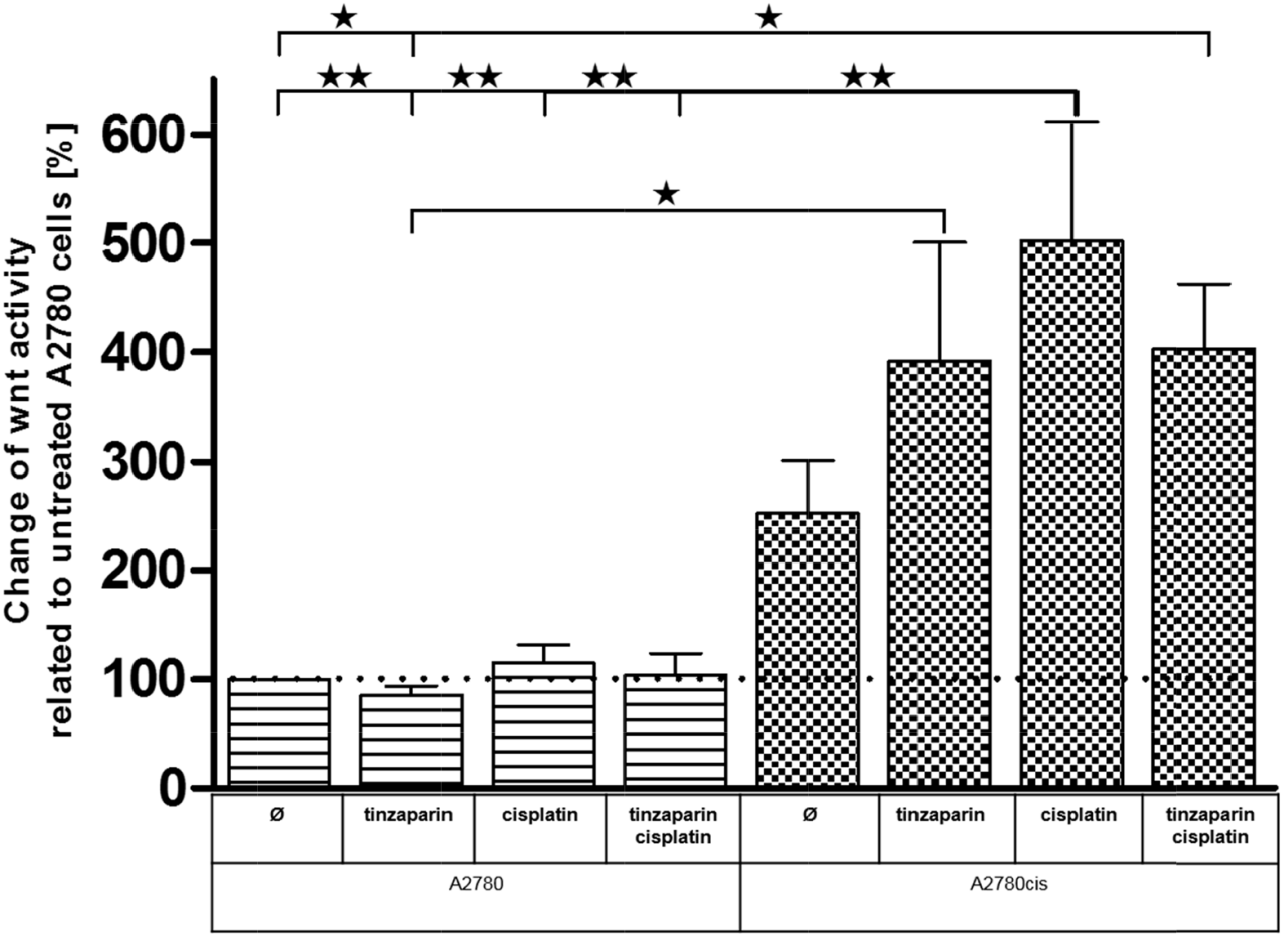
Wnt activity of A2780 and A2780cis cells Cells were either untreated or preincubated with tinzaparin 24 h before cisplatin addition. Luciferase activity was analyzed 72 h after treatment by luminescence measurement and normalized to untreated A2780 cells. Data from three independent experiments.

To confirm a probably higher dependency of A2780cis cells on Wnt activity, and to illustrate a direct link between LMWH activity in sensitizing the cells for cisplatin with the Wnt pathway, we transfected the cells with a TCF/LEF luciferase-coupled promotor vector. First we assured that the transfection did not change the resistance, both, the transfected A2780 and A2780cis cells displayed an identical behavior like the non-transfected counterparts and the resistance factor was maintained (Supplementary Figure 1). The luciferase measurements (Figure 7) clearly indicate that in the untreated state, A2780cis cells display a nearly threefold higher Wnt activity than A2780 cells. This low activity in A2780 cells is hardly affected by cisplatin, tinzaparin or both components combined, indicating that this signaling pathway is of low relevance in these cells. In strict contrast, cisplatin induces a massive upregulation and doubling of the even high Wnt activity in A2780cis cells. Tinzaparin reduces this activity when combined with cisplatin. However, the increase in Wnt activity by tinzaparin alone is unexpected and will be discussed elsewhere.

### Tinzaparin interferes with TCF-4 expression

**Figure 8:**
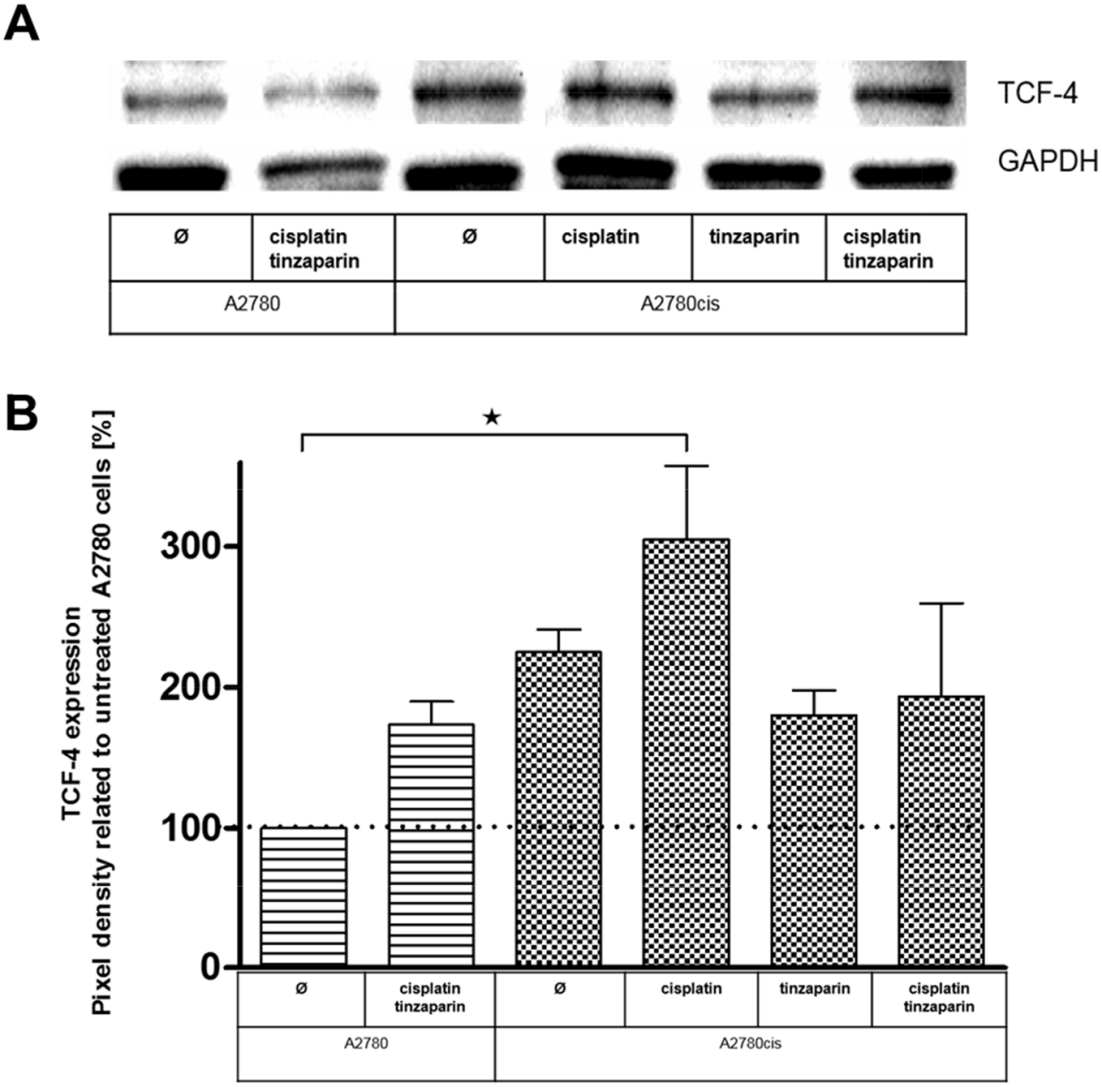
**(A)** Expression of TCF4 in A2780 and A2780cis cells with a 24 h tinzaparin preincubation before cisplatin addition. Expression was analyzed 72 h after cisplatin treatment by immunoblotting using a specific antibody and shown as blot for one representative experiment and as relative expression by pixel density measurements related to untreated A2780 cells **(B)**. Data from three independent experiments.

As a further insight into the transcriptional activity of the Wnt pathway we focused on TCF-4 at the protein level by western blots. As illustrated in Figure 8, A2780cis cells have a more than duplicated amount of this transcription factor in the untreated state, which is in total agreement with the luciferase activity data in Figure 7. Furthermore it revises the unexpected finding of lower TCF-4 at the mRNA level in Figure 4.

Cisplatin increases the TCF-4 expression in A2780cis cells indicating that Wnt pathway upregulation is indeed a clear response to cisplatin treatment in the resistant cells. Tinzaparin possesses an even more evident inhibition of this cisplatin induced upregulation than in case of the luciferase data, turning the expression of TCF-4 even under the level of the untreated A2780cis cells.

## DISCUSSION

Here we provide evidence that the Wnt signaling pathway is the dominant mechanism driving cisplatin resistance in A2780cis cells and thus appears as primary target for LMWH in sensitizing the cells. Furthermore, our data suggest that heparanase has no obvious contribution to A2780cis resistance in the untreated state. Despite the lower expression in response to tinzaparin and cisplatin together, this enzyme appears not likely as target for heparin in these terms, at least not under the 2D *in vitro* cultivation of these cells. Nevertheless, the attenuated heparanase expression by the combined treatment in A2780 cells could have a certain impact on the Wnt signaling pathway, reducing the cleavage of bound Wnt ligands from HSPG. The independence of resistance on cell-ECM contacts makes adhesion-mediated resistance phenomena, and thus an interference of LMWH with integrin binding also unlikely.

Canonical Wnt signaling is a major pathway in various tumor entities, including ovarian cancer [37] driving tumorigenicity on various functional axes and appears as attractive target for therapeutic approaches [38].

HSPG are critical control elements of the Wnt signaling pathway, which bind Wnt ligands and regulate their access to the signal transduction receptors at the cell surface. We demonstrate here by ^35^Sulphur radio labeling experiments that A2780 and A2780cis cells differ in their HSPG structure, namely the resistant cells express HS structures with slightly higher chain length and higher charge intensities. These findings are reflected by the expression levels of the 6-O-sulfatase Sulf-2, which displayed a slightly lower level in A2780cis cells than in A2780 cells. Although the overexpression of the 6-O-sulfatases Sulf-1 and / or 2, which remove 6-O sulfates from the N-glucosamines of the HS fractions have been described to intensify Wnt pathway activity and oncogenic effects in human pancreatic tumor cells [39], hepatocellular carcinomas [40] or human prostate cancer cells [41], respectively, contrary findings also exist. Sulf 1 has been described to inhibit the transcriptional activity in the Wnt β-catenin pathway in other tumor entities, such as in gastric cancer cells [42].

A probably more direct link between HSPG and Wnt activity as reason for cisplatin resistance is given by our GPC3 expression data. The role of this HSPG for intensifying the Wnt signaling is known for many years [24]. Recently, Capurro et al. described a direct role of GPC3 in the contact formation with Frizzled which goes beyond the passive accumulation of Wnt ligands at the cellular HSPG for receptor access [35]. Interestingly, the slightly lower GPC3 expression in A2780cis cells is massively upregulated after 72 h when pretreated with tinzaparin. GPC3 upregulation has also been associated with an intensified fibroblast growth factor signaling pathway in hepatocellular carcinomas [43]. However, Lai et al. figured out an FGF/GPC3 activation axis on the basis of upregulated Sulf-2 in these carcinomas. Since Sulf-2 was even lower expressed in our resistant cells, we did not follow the FGF activation path and rather focused on Wnt signaling.

It seems likely that LMWH, based on its GAG structure interferes with the HSPG activities at the cell surface, such as the GPC3 interaction with Wnt ligands and Frizzled. Consequently, A2780cis cells try to maintain the Wnt activity and to antagonize this inhibitory effect by upregulating GPC3 as an auto-regulatory loop. This hypothesis is further supported by the finding of increased HS chain length and total amount in the A2780cis cells, which may have contributed to stabilization of Wnt-receptor complex.

A similar interpretation of an auto-regulatory induced, primary increase in Wnt activity by the interference with LMWH can also be derived from the TCF/LEF activity luciferase measurements in Figure 7. Before coming to that point, the luciferase data in general indicate clearly that the resistant cells have an up to threefold higher Wnt activity compared to A2780 cells. The link between cisplatin resistance in A2780cis cells and Wnt is supported by recent findings of Zhao et al. [44] who reported on higher nuclear localization of β-catenin in resistant A2780 cells. In contrast to the resistant cells, neither cisplatin nor tinzaparin nor the combination of both induces remarkable changes in the Wnt activity of A2780 cells. This suggests that Wnt is of minor importance in A2780 cells, which is also supported by the MTT data. In resistant cells, cisplatin induces a strong increase of the even higher TCF/LEF activity, which indicates that cytotoxic stress is answered by the cells with upregulating Wnt pathway activity. However, tinzaparin can diminish this upregulation, but tinzaparin pretreatment alone also resulted in higher luciferase activity, which appears in line with the GPC3 upregulation, mentioned before and the increase in TCF-3 (TCF7L1) mRNA in Figure 4. The obvious Wnt activation by tinzaparin alone in A2780cis cells appears contradictory, since we are searching for the inhibitory mechanism of LMWH interference. One can suggest that in absence of cisplatin, tinzaparin deregulates HSPG driven processes at the cell surface, such as Wnt signaling by interfering with the Wnt ligand binding to HSPGs and receptors. Thus the cells maintain the higher intrinsic Wnt activity by a counter regulation. Cisplatin alone appears as a clear trigger for Wnt activity in A2780cis cells and as a main reason for resistance. When induced by cisplatin, the high Wnt signaling activity will be disturbed and inhibited by LMWH. The dominance of the inhibitory mechanism of the LMWH interference becomes evident by the TCF-4 blotting data in Figure 8. Interestingly, A2780cis cells display more than a twofold higher expression of this transcription factor than A2780 cells which reflects the differences in luciferase activity quite well. Cisplatin even increases TCF-4 expression, which also refers to the activity data. LMWH in turn clearly blocks the intrinsic and the cisplatin-induced TCF-4 expression even under the level of A2780cis cells. This explains the functional readout using cytotoxicity assays in our recent study, indicating similar IC_50_ values of A2780 and A2780cis cells for cisplatin cytotoxicity when treated with tinzaparin.

Concluding, although we cannot exclude whether tinzaparin targets other processes in sensitizing the A2780cis cells for cisplatin than the Wnt pathway, we selected important key players of the Wnt pathway that should allow the implication that targeting of Wnt signaling appears as a primary activity of LMWH.

In summary, concluding our data we provide evidence that LMWH impacts the Wnt signaling pathway that is a major component for cisplatin resistance in A2780cis cells. Therefore, LMWH is able to return this resistance to the level of A2780 cells. Our data underline the functional findings we published before and add a novel mode of action to the therapeutic heparin application in oncology referring a potential interference with chemoresistance. Further studies are needed to investigate whether these findings are relevant to other chemoresistant cell lines *in vitro* and if they are also applicable under *in vivo* conditions.

## MATERIALS AND METHODS

### Cell culture

A2780 (No. 93112519) and the cisplatin resistant A2780cis (No. 93112517) human ovarian carcinoma cell lines were purchased from the ECACC, UK and cultivated by 37°C and 5% CO_2_ in RPMI 1640 medium containing 10% FCS, 1% penicillin/streptomycin (PAN Biotech, Aidenbach, Germany) and 1.5% L-glutamine. Upon receipt, cells were directly frozen in aliquots (master cell bank) from which they were cultivated for a maximum of ten passages for functional studies and gene expression profiling. Authenticity of cells was confirmed by short tandem repeat (STR) profiling. The maintenance of cisplatin resistance in A2780cis cells as well as the absence of mycoplasma in cell culture was confirmed every second week.

### Metabolic labeling, purification and analysis of heparan sulfate

A2780 and A2780cis cells were cultured in 75 cm^2^ flasks to 80% confluence. After changing to fresh medium (8 mL RPMI per flask) 100 μCi × mL^−1^ of Na_2_^35^SO_4_ (Perkin Elmer, Mechelen, Belgium) was added and cells were cultured for 24 h. Medium was collected, cells were washed with DPBS and lysed using 50 mmol × L^−1^ Tris-HCl pH 7.4, 1% Triton X-100.

After centrifugation at 15,800 g, 4°C for 10 min, the supernatants were used for purification of HS by ion exchange chromatography. Medium and supernatant of cell lysates were applied to a 1 mL DEAE-Sephacel column (GE Healthcare Biosciences, Uppsala, Sweden) pre-equilibrated with 50 mmol × L^−1^ Tris HCl pH 7.4, 1% Triton X-100. The columns were washed with 50 mmol × L^−1^ Tris-HCl pH 7.4 followed by 50 mmol × L^−1^ NaAc pH 4.5, 0.25 mol × L^−1^ NaCl, and negatively charged proteoglycans were eluted with 50 mmol × L^−1^ NaAc pH 4.5, 2 mol × L^−1^ NaCl. Eluted material was desalted on a PD-10 column (GE Healthcare Biosciences, Uppsala, Sweden) followed by lyophilization to dryness. The samples were then treated overnight at 37°C with 0.2 U chondroitinase ABC per sample (Seikagaku, Tokyo, Japan) to degrade chondroitin sulfate (CS). Complete digestion by chrondroitinase ABC was assured by complete degradation of the samples with HNO_2_ at pH 1.5 followed by size analysis chromatography.

Heparan sulfate chains were released from the core proteins by alkali treatment in 0.5 mol × L^−1^ NaOH overnight on ice. The samples were applied to a Superose 12 column (GE Healthcare Biosciences, Uppsala, Sweden), equilibrated with 50 mmol × L^−1^ HEPES pH 7.4, 1 mol × L^−1^ NaCl or a Mono Q column (GE Healthcare Biosciences, Uppsala, Sweden), equilibrated with 50 mmol × L^−1^ NaAc pH 4.5, 0.25 mol × L^−1^ NaCl for assessment of molecular size or overall charge density, respectively. The eluates were collected as 0.5 mL fractions and ^35^S-radioactivity was analyzed by mixing the samples with scintillation cocktail and β-scintillation counting on a Beckman Coulter instrument (Perkin Elmer, Upplands Väsby, Sweden).

### Determination of cisplatin and FH535 cytotoxicity by MTT assays

Cytotoxicity of the Wnt-inhibitor FH535 and cisplatin in A2780 and A2780cis cells was determined by MTT (3-(4,5-dimethylthiazole-2-yl)-2,5-diphenyltetrazolium bromide)-assay 72 h after cisplatin addition as described [17].

FH535 was used in concentrations ranging from 10^−4.26^ to 10 ^−8.76^ mol × L^−1^ and for cisplatin from 10^−3.5^ to 10^−7.5^ mol × L^−1^. 20,000 and 40,000 cells were seeded in a total volume of 100 μL per well. Cisplatin cytotoxicity was determined using cells that were pretreated for 1.5 h with FH535 at a concentration of 0.5 μmol × L^−1^ or cells were seeded 24 h before cisplatin addition in collagen coated wells (10 μg × cm^−2^). In other experiments, A2780 and A2780cis cells were treated with roneparstat at concentrations of 5 and 10 μmol×L^−1^ 5 h before addition of cisplatin.

### Immunoblotting

Expression of the proteoglycan glypican-3 (GPC3), the transcription factor TCF-4 and heparanase were analyzed by SDS-PAGE and immunoblotting. Therefore, A2780 and A2780cis cells were treated with tinzaparin (50 μg × mL^−1^) 24 h before addition of cisplatin (2 μmol × L^−1^) and after 72 h lysed with cell extraction buffer (Life Technologies, Darmstadt, Germany). Mouse anti-GPC3, a rabbit anti-TCF-4, a mouse anti-Sulf-2 and a rabbit anti-heparanase antibody were used to detect the proteins. Immunoblots were developed by using specific HRP-conjugated secondary antibodies. Luminol reaction was detected by the ChemiDoc XRS+ System (Bio-Rad Laboratories, Munich, Germany). Pixel densities of detected proteins were normalized to the housekeeping protein GAPDH.

### FACS analysis of syndecan-1, syndecan-4 and glypican-3, glypican-4

Quantification of proteoglycans was performed using a FACSCalibur (BD, Heidelberg, Germany). For detection, rabbit anti-SDC-1, rabbit anti-SDC-4, mouse anti-GPC3 and mouse anti-GPC4 antibodies were used. The secondary goat anti-rabbit and goat anti-mouse antibodies against SDC-1, SDC-4, GPC3 and GPC4 were FITC labeled.

### Luciferase reporter assay

One day before transfection, A2780 and A2780cis cells were seeded in a 24-well plate in 1 mL cell culture medium. At transfection procedure, cells were about 50% confluent and they were transfected with pGL4.49[luc2P/TCF-LEF/Hygro] and FuGENE^®^ transfection reagent following manufacturer’s protocol. Two days later, transfected cells were selected by changing to cell culture medium containing 100 μg × mL^−1^ hygromycin (Invivogen, Toulouse, France). Cells were frozen at −80°C until luminescence measurements were performed. Therefore, 40,000 cells were seeded in a white 96-well plate in a total volume of 100 μL. Luminescence intensity was determined using cells that were treated 24 h with tinzaparin (50 μg×mL^−1^) before addition of cisplatin (2 μmol × L^−1^). Luciferase activity was analyzed by adding 100 μL ONE-Glo™ Luciferase Assay System to each well. After 5 minutes of lysis, luminescence intensity was detected using a FLUOstar Optima (BMG Labtech, Ortenberg, Germany). Values were normalized to total protein concentration using Pierce™ BCA Protein Assay Kit (Thermo Fisher Scientific, Waltham, USA) following manufacturer’s instructions.

### Preparation of RNA and microarray analysis

Cell lysis, extraction of RNA, microarray data analysis and processing of microarray raw data were performed as described before [17] and [45].

### Statistics

Cytotoxicity data of the sigmoidal dose-response curves were evaluated by a nonlinear regression using the four-parameter logistic equation with variable hill slope (GraphPad Prism 5.0 software, San Diego, USA). Graphical data represent means ± SEM (standard error of the mean) of a representative experiment performed in triplicates. Comparison of the microarray data collective, western blot and luciferase reporter data was performed by one way ANOVA following Tukey’s multiple comparison test. The star (double-star) symbol represents *p*-values smaller than 0.05 (0.01).

### Materials

MTT (3-(4,5-dimethylthiazole-2-yl)-2,5-diphenyltetrazolium bromide) was purchased from Sigma–Aldrich (Steinheim, Germany), the transfection vector pGL4.49[luc2P/TCF-LEF/Hygro], FuGENE^®^ HD Transfection Reagent and ONE-Glo™ Luciferase Assay System from Promega (Madison, USA). The heparanase inhibitor roneparstat was kindly provided by Sigma-tau Research (Mendrisio, Switzerland), LMWH tinzaparin was from LEO-Pharma (Neu-Isenburg, Germany), collagen was purchased from Roche (Mannheim, Germany) and the Wnt-pathway inhibitor FH535 from Sigma-Aldrich (Steinheim, Germany).

Anti-SDC-1, -SDC-4, -GPC3, -Sulf-2 and -TCF-4 antibodies were obtained from Santa Cruz Biotechnologies (Heidelberg, Germany) and anti-GPC4, -heparanase and -GAPDH from GeneTex (Irvine, USA). ECL solution, streptactin and Precision Plus Protein™ Unstained Protein Standard were purchased from Bio-Rad, Munich, Germany. All salts and buffer used were of analytical grade.

## SUPPLEMENTARY MATERIALS FIGURE



## Abbreviations

CAM-DR: Cell adhesion mediated drug resistance
Cpm: counts per minute
CS: chondroitin sulfate
ECM: Extracellular matrix
GAG: glycosaminoglycan
GPC3: glypican-3
GPC4: glypican-4
HSPG: Heparan sulfate proteoglycan
HS: heparan sulfate
LMWH: Low molecular weight heparin
Rf: resistance factor
SDS: sodium-dodecylsulfate
SDC-1: syndecan-1
SDC-4: syndecan-4
