# Supplementary material for: Heparin antagonizes cisplatin resistance of A2780 ovarian cancer cells by affecting the Wnt signaling pathway
Source: Oncotarget. 2017 Jun 28;8(40):67553–66. doi: 10.18632/oncotarget.18738 (PMC5620193; doi:10.18632/oncotarget.18738)
Supplement: Supplementary file 1 [file oncotarget-08-67553-s001.pdf]

Heparin antagonizes cisplatin resistance of A2780 ovarian cancer cells by affecting the Wnt signaling pathway

SUPPLEMENTARY MATERIALS

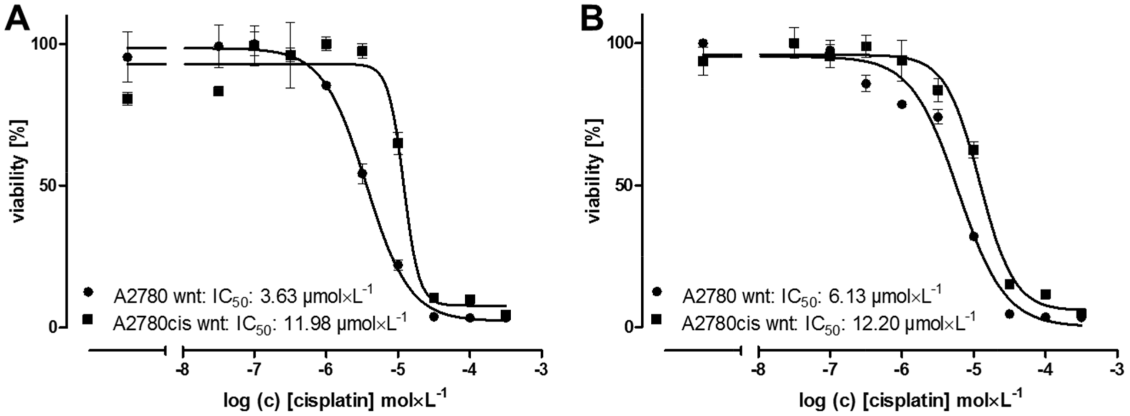

Supplementary Figure 1: Determination of cytotoxicity 72 h after addition of cisplatin displayed as IC_{50} in luciferase transfected A2780 (circles) and A2780cis (squares) cells by MTT assays. The IC_{50} for cisplatin are indicated in the figure for a representative experiment. Cells were seeded in a count of 20,000 **(A)** and 40,000 **(B)** 24 h before treatment with cisplatin. MTT assays were performed in triplicates.
